# The frequency and clinicopathological significance of *NRAS* mutations in primary cutaneous nodular melanoma in Indonesia

**DOI:** 10.1002/cnr2.1454

**Published:** 2021-06-10

**Authors:** Hanggoro Tri Rinonce, Deflen Jumatul Sastri, Fita Trisnawati, Bidari Kameswari, Paranita Ferronika, Irianiwati

**Affiliations:** ^1^ Department of Anatomical Pathology, Faculty of Medicine, Public Health, and Nursing Universitas Gadjah Mada/ Dr. Sardjito Hospital Sleman Yogyakarta Indonesia; ^2^ Department of Anatomical Pathology dr. Soeradji Tirtonegoro Hospital Klaten Central Java Indonesia

**Keywords:** Indonesia, nodular melanoma, *NRAS* mutation, RAS gene family, skin tumor

## Abstract

**Background:**

Melanoma is a lethal skin malignancy with a high risk of metastasis, which prompts a need for research on treatment targets and prognostic factors. Recent studies show that the presence of neuroblastoma RAS viral oncogene homolog (*NRAS*) mutation can influence cell growth in melanomas. The *NRAS* mutation, which stimulates the mitogen‐activated protein kinase (MAPK) signaling pathway, is associated with a lower survival rate. However, evidence from Indonesia population is still very rare. Further understanding of the role of *NRAS* mutations in Indonesian melanoma cases will be crucial in developing new management strategies for melanoma patients with *NRAS* mutations.

**Aims:**

To explore the frequency of *NRAS* mutations and their clinicopathological associations in patients with primary nodular cutaneous melanoma in Central Java and Yogyakarta, Indonesia.

**Methods and results:**

Fifty‐one paraffin‐embedded tissue samples were collected from primary nodular skin melanoma cases between 2011 and 2019 from the two largest referral hospitals in Yogyakarta and Central Java, Indonesia. The *NRAS* mutation status was evaluated using qualitative real‐time polymerase chain reaction (qRT‐PCR). The association of *NRAS* mutation was analyzed with the following: age, gender, location, lymph node metastasis, ulceration, mitotic index, tumor‐infiltrating lymphocytes (TILs), necrosis, tumor thickness, lymphovascular invasion (LVI), and tumor size. *NRAS* mutations were detected in 10 (19.6%) samples and predominantly observed (60%) in exon 2 (G12). These mutations were significantly correlated with lymph node metastases (*p* = .000); however, they were not associated with other variables analyzed in this study.

**Conclusions:**

The prevalence of *NRAS* mutations in primary nodular cutaneous melanoma cases from Indonesia is consistent with previous studies and is significantly associated with increased lymph node metastases. However, the predominant mutation detected in exon 2 (G12) is different from previous studies conducted in other countries. This suggests that melanoma cases in Javanese people have different characteristics from other ethnicities.

## 1 INTRODUCTION

Although considered rare compared with basal and squamous cell carcinomas, melanoma is a lethal skin malignancy that has a high risk of metastasis. The incidence rate of skin melanoma varies throughout the world. In western countries, melanoma is relatively frequent, especially in light‐skinned populations. Recent research shows that the highest incidences are in Queensland, Australia, and Auckland, New Zealand.^1^ The incidence of melanoma is low in Asian populations, which is approximately 0.25 per 100 000 population in 2018, according to the Global Cancer Observatory.^2^

The prognosis of cutaneous melanoma is influenced by several clinical and histopathological factors. Clinical factors include age, sex, and anatomic location. Histopathological factors consist of Breslow tumor thickness, ulceration, Clark's anatomic level, tumor volume, growth patterns, mitosis number, radial and vertical growth phase, regression, tumor vascularity, lymphovascular invasion (LVI), angiotropism, histological type, and tumor‐infiltrating lymphocytes (TILs).^3^

Recent research shows that cell growth in melanomas is also influenced by the *NRAS* gene. The presence of *NRAS* mutations stimulates the *RAS*‐*RAF*‐extracellular signal‐regulated kinase/mitogen‐activated protein kinase (*MAPK*) signaling pathways that can interfere with cell cycle regulation and prosurvival pathways and increase cell proliferation.^4^ *NRAS* mutations occur in 15%‐25% of melanoma cases, most often in exons 2 (codon 12) and 3 (codon 61). The presence of *NRAS* mutations was associated with a lower survival rate.^5^

Until now, no prognostic marker has been validated for melanoma. Further understanding of melanoma with *NRAS* mutations will be crucial in developing new management strategies for melanoma patients with *NRAS* mutations.^6^

The study of cutaneous nodular melanoma in Asia has been scarce because of its uncommon nature. Although the frequency is low, nodular melanoma is a major contributor to skin cancer‐related mortalities. One study conducted in Australia found nodular melanomas represented 14% of the invasive melanomas and shockingly caused 43% of the deaths.^7^ This poor outcome implores further elucidation on this particular type of melanoma.

Further research concerning *NRAS* mutations and its associations is needed because of the lack of evidence in Indonesia. This study aimed to examine the frequency of *NRAS* mutations and their associations with clinicopathological properties in patients with primary nodular cutaneous melanoma in Central Java and Yogyakarta, Indonesia.

## 2 METHODS

A retrospective cross‐sectional study was conducted in the Department of Anatomical Pathology Dr. Sardjito Hospital, Sleman, Yogyakarta and Dr. Soeradji Tirtonegoro Hospital, Klaten, Central Java. Both hospitals are the major referral hospitals in Yogyakarta and Central Java Province, Indonesia. We collected and analyzed 51 paraffin‐embedded tissue samples from primary nodular cutaneous melanoma cases from 2011 to 2019. The ethnicity of the patients was Javanese, which is one of the major ethnic groups in Indonesia.

The existence of *NRAS* mutation was examined using qualitative real‐time polymerase chain reaction (qRT‐PCR). The DNA source was collected from four slices (5 μm thickness) of formalin‐fixed paraffin‐embedded tumor tissues. Slides were examined under a microscope after deparaffinization and hematoxylin‐eosin staining. DNA was extracted from the tumor‐containing areas using GeneAll Exgene DNA Extraction Kit (GeneAll Biotechnology, Seoul, Korea) according to the instructions of the manufacturer. DNA amplification was performed using the AmoyDx *NRAS* Mutations Detection Kit (AmoyDx, Xiamen, China). The qRT‐PCR was used to detect 16 hotspot somatic mutations in codons 12 and 13 (exon 2), 59 and 61 (exon 3), and 117 and 146 (exon 4) of the *NRAS* gene. The NRAS positive control solution from the kit was used for positive control, while distilled water was used as a negative control.

The clinicopathological data, including age, sex, anatomic location, lymph node metastasis, ulceration, mitosis number, TILs, necrosis, tumor thickness, LVI, and tumor size, were collected. Hematoxylin‐eosin stained slides were examined under a microscope for ulceration, tumor thickness, lymph node metastasis, LVI, necrosis, and TILs. The existence or lack of lymph node metastasis was evaluated by examining the lymph node biopsy. Tumor thickness was estimated from the granular layer to the deepest invasion of the tumor and subsequently categorized as ≤4 or >4 mm. Ulceration was defined as the combination of a full‐thickness epidermal defect (including the absence of stratum corneum and basement membrane), evidence of host response (i.e., fibrin deposition and neutrophils), and thinning, effacement, or reactive hyperplasia of the surrounding epidermis.^8^ LVI was defined as the presence of tumor cells within the blood vessel and/or lymph vessel, encasing the tumor identical to the primary cutaneous melanoma cells. Necrosis was defined as the existence of an area of necrotic cells occupying at least one‐fourth high‐power field (0.07 mm^2^). TILs were defined as migrating lymphocytes from the blood vessels to the surrounding tumor stroma and categorized into present or absent. Non‐brisk and brisk TILs are classified into present category. Tumor size was measured from the length of the larger axis in millimeters after formalin tissue fixation.^9^, ^10^

Immunohistochemistry was performed using a manual method from ScyTek Laboratories (ScyTek Laboratories Inc., Utah, USA). In this study, we used the CellMarque Ki‐67 (SP6) monoclonal antibody (Sigma‐Aldrich Co., Oakville, Canada). Tonsil tissue slides were used as a positive control. Paraffin blocks were sliced as thick as 3 μm, incubated, deparaffinized, and rehydrated. Antigen retrieval was conducted using Tris EDTA solution (Vivantis Inc., California, USA) at pH 9 for 20 min at 95°C. Slides were submerged for 15‐20 min in 3% hydrogen peroxide, soaked in primary antibody for 60 min, labeled with horseradish peroxidase polymer for 10‐20 min and diaminobenzidine for 3 min. Finally, slides were counterstained using hematoxylin for 0.5‐1 min and then covered by the coverslips. The mitotic index was counted based on the percentage of positively stained nuclei per 1000 tumor cells and then categorized as <20% and ≥20%. The associations between *NRAS* mutation status and clinicopathological parameters were analyzed using the chi‐squared or Fisher's exact tests.

## 3 RESULTS

The subjects' mean age was 63 years, ranging from 21 to 95 years. Of patients, 17 (33%) were men and 34 (67%) women. The tumors were found in the extremities in 41 subjects (80%), whereas the rest were in the trunk, head, and neck (centrally located). Out of 51 subjects, *NRAS* mutations were seen in 10 (19.6%) samples, and of these, mutations in exons 2 (G12) and 3 (Q61) were detected in 6 (60%) and 4 patients (40%), respectively.

**FIGURE 1:**
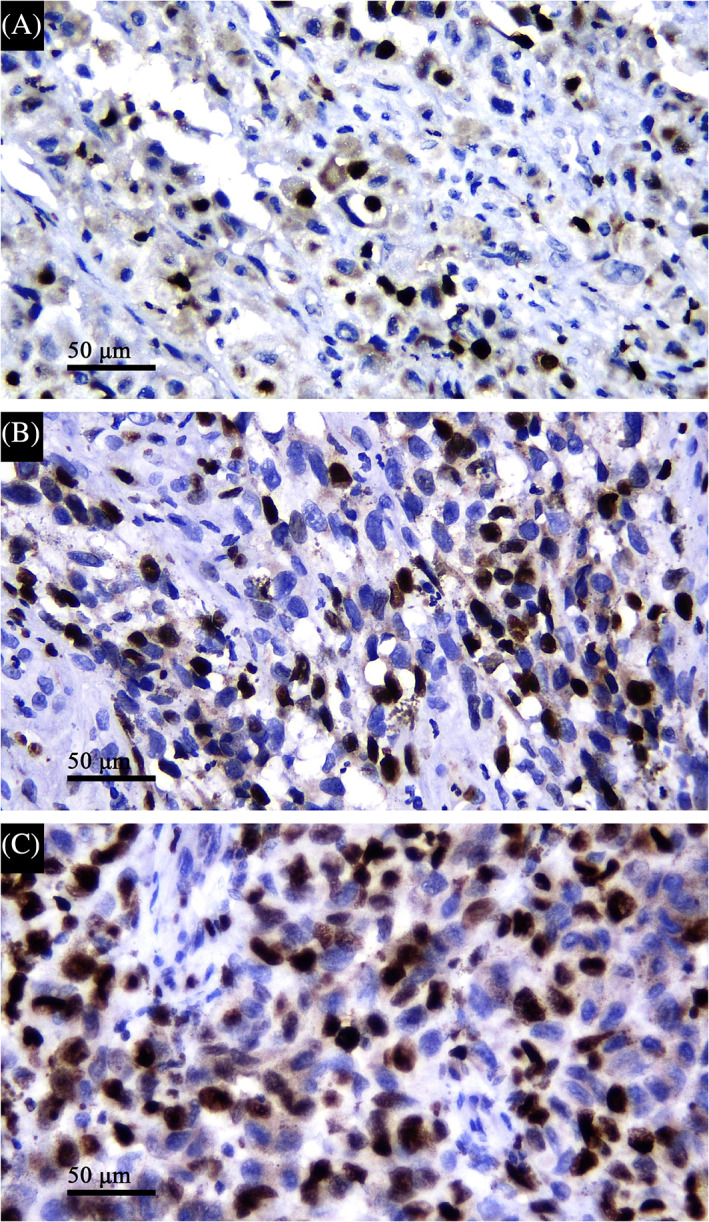
Representative results of Ki‐67 immunohistochemical staining with 400× magnification of the tissue slides with varying mitotic indexes: 8% (A), 23% (B), and 41% (C). Scale bar represents 50 μm

The association between *NRAS* mutation and the clinicopathological parameters is shown in Table 1. Representative results of Ki‐67 proliferative index measurement are shown in Figure 1. *NRAS* mutation was highly correlated with lymph node metastasis (*p* = .000). No significant association was found between *NRAS* mutation and other variables analyzed in this study.

**TABLE 1:** The association between *NRAS* mutation and clinicopathological parameters

	*NRAS* (+)	*NRAS* (−)	*p* value^†^
Age category (years), *n* (%)			
≤65	6 (12)	20 (39)	.726
>65	4 (8)	21 (41)	
Sex, *n* (%)			
Male	3 (6)	14 (27)	1.000
Female	7 (14)	27 (53)	
Anatomic location, *n* (%)			
Extremity	9 (18)	32 (63)	.664
Central	1 (2)	9 (17)	
Lymph node metastases, *n* (%)			
Present	10 (20)	15 (29)	**.000** ^†^
Absent	0 (0)	26 (51)	
Tumor thickness (mm), *n* (%)			
≤4	0 (0)	7 (14)	.320
>4	10 (20)	34 (66)	
Ulceration, *n* (%)			
Present	5 (10)	26 (51)	.586
Absent	5 (10)	15 (29)	
Mitotic index category, *n* (%)			
≥20%	6 (12)	19 (37)	.499
<20%	4 (8)	22 (43)	
Necrosis, *n* (%)			
Present	9 (18)	23 (45)	.069
Absent	1 (2)	18 (35)	
Lymphovascular invasion, *n* (%)			
Present	3 (6)	11 (21)	1.000
Absent	7 (14)	30 (59)	
Tumor‐infiltrating lymphocytes, *n* (%)			
Present	8 (16)	31 (60)	1.000
Absent	2 (4)	10 (20)	
Tumor size (mm)			
≤6	3 (6)	16 (31)	.725
>6	7 (14)	25 (49)	

*Note*: Significant values are shown in bold.

^†^ A *p*‐value < .05 was defined as significant.

## 4 DISCUSSION

Melanoma incidence in the last 50 years has increased significantly from 8.2 to 9.4 cases per 100 000 population in 1975 to 24.2 to 35.4 cases in 2010 in the United States.^11^ The mortality rate caused by melanoma is high, approximately 79% in the United States.^12^ Of the total 70 230 patients diagnosed with skin melanoma in 2011, 8790 died from the disease.^5^

The prognosis of melanoma patients is influenced by several clinical and histopathological factors. Clinical factors include age, sex, and anatomic location, whereas histopathological factors consist of tumor thickness, ulceration, anatomic level, LVI, growth pattern, tumor volume, mitosis number, histological subtype, regression, radial and vertical growth phase, tumor vascularity, angiotropism, and TILs.^3^

In this study, *NRAS* mutation was observed in 19.6% of subjects. This result is consistent with previous studies showing that *NRAS* mutation rates in skin melanoma cases in Asia, Europe, Africa, Australia, and America ranged from 10% to 26% (Table 2). We found that the *NRAS* mutation prevalence was evidently higher than the *BRAF* mutation in Indonesia.^29^ Previous studies demonstrated that *NRAS* mutations occur more commonly in nodular subtype melanomas than in other subtypes and often develop in the skin with continuous ultraviolet exposure.^20^

**TABLE 2:** Summary of *NRAS* mutation in primary or metastatic melanoma worldwide

Continent and references	Country	*NRAS* mutation prevalence (*n* positive/*n* total [%])	Mutation profile		Method	Melanoma
			Exon 2 (G12 or G13) [%]	Exon 3 (Q61) [%]		
Asia						
Sheen et al^13^	Taiwan	12/119 (10.1)	16.7	83.3	Sanger sequencing	Cutaneous
Choi et al^14^	Korea	0/22 (0)	0	0	Sanger sequencing	Mucosal and cutaneous
Sakaizawa et al^15^	Japan	21/171 (12.3)	NA	76.2	Sanger sequencing	Mucosal and cutaneous
Yilmaz et al^16^	Turkey	10/47 (21.3)	0	100	Sanger sequencing	Cutaneous
Lyu et al^17^	China	0/57 (0)	0	0	Sanger sequencing	Oral mucosal
Uhara et al^18^	Japan	9/127 (7.1)	22.2	77.8	Sanger sequencing	Mucosal and cutaneous
Si et al^19^	China	31/432 (7.1)	29	58	Sanger sequencing	Mucosal and cutaneous
Present study (2020)	Indonesia	10/51 (19.6)	60	40	RT‐PCR	Cutaneous
Europe						
Heppt et al^20^	German	53/217 (24.4)	5.6	86.8	Sanger sequencing	Mucosal and cutaneous
Van Engen‐Van Grunsven et al^21^	Netherlands	4/24 (16.6)	25	75	Sanger sequencing	Female urogenital mucosal
Zebary et al^22^	Sweden	8/56 (14.3)	50	50	Sanger sequencing	Sinonasal mucosal
Colombino et al^23^	Italy	15/102 (14.7)	0	100	Sanger sequencing	Cutaneous
Manrique‐Silva et al^24^	Spain	65/563 (11.5)	13.8	72.3	Sanger sequencing	Cutaneous
America						
Jakob et al^5^	USA	136/677 (20.1)	17.6	82.4	Sanger sequencing	Mucosal and cutaneous
Goel et al^25^	USA	10/60 (16.7)	0	100	Sanger sequencing	Cutaneous
Africa						
Akslen et al^26^	Tanzania	26/118 (22.0)	19.2	80.8	Sanger sequencing	Cutaneous
Australia						
Jones et al^27^	New Zealand	124/466 (26.6)	7.3	92.7	Sanger sequencing	Mucosal and cutaneous
Carlino et al^28^	Australia	39/193 (20.2)	15.4	84.6	Sequenom OncoCarta Panel	Metastatic

Abbreviations: NA, not available data; RT‐PCR, real‐time polymerase chain reaction.

In this study, all tumor samples were from Asian races, specifically Javanese ethnicity, which is the most common ethnic group in Indonesia. Since Indonesia is located on the equator, high‐intensity sunlight is continuously present throughout the year, which may cause skin damage because of the high cumulative sun damage (CSD), particularly in older people. Melanomas that arise due to sun exposure are generally dominated by *NF1* and *NRAS* mutations.^30^, ^31^ The sun exposure is also related to the acral lentiginous type of melanoma, which generally has a thin tumor thickness.^32^

This study also showed that *NRAS* mutation was predominantly present in exon 2 (G12). This finding contradicts some previous studies that demonstrated that the predominant site of *NRAS* mutation is in exon 3 (Q61).^5^, ^25^, ^33^ This discrepancy might be due to differences in the characteristics of the sample population used among different studies. Bucheit et al reported that *NRAS* exon 2 (G12) mutations are more common in mucosal melanomas and *NRAS* exon 3 (Q61) mutations in nodular and superficial spreading melanomas.^33^ The Bucheit study sample numbered 136, but only 58 (42%) samples were a combination of nodular type melanoma and superficial spreading, and 50 samples (86%) were with mutations in exon 3 (Q61).^33^ In this study, we used samples from nodular type melanoma only.

In this study, we found a significant association between lymph node metastasis and *NRAS* mutation. This finding is in concordance with the results of the study by Sheen et al that revealed that melanoma with *NRAS* mutations has the nature and pattern of aggressive growth. This group of melanoma tends to develop lymph node and even distant metastases.^13^ Sentinel lymph node (SLN) biopsy is a mandatory procedure to achieve sufficient data about lymph node metastasis even with a small number of samples. SLN positivity itself has been accepted as a prognostic factor for tumor recurrence and poor prognosis.^34^ *NRAS*‐mutant tumors are likely to act aggressively, especially in the early stages in the high‐risk melanoma population. In our study, 45 (88%) patients had lymph node metastasis. Accordingly, most of the patients had a higher risk of recurrence and poor prognosis. All patients with *NRAS* mutation had lymph node metastasis, proving that this mutation has an important role in more aggressive tumor behavior.

We found a predominance of female patients in our study. Some studies show that women who suffer from melanoma have a good prognosis, even patients with lymph node metastasis. The gender influence in the prognosis of the disease is related to differences in thickness, ulceration, and anatomic location of the melanoma between women and men. The presentation of melanoma is thinner, and ulceration is less common in women than in men.^35^ Thicker tumors tend to have higher mitotic rates.^36^

This study demonstrated no statistically significant association between *NRAS* mutations and anatomic locations. In a previous study by Sheen et al in Taiwan with 119 samples, the extremities were the most common location for melanomas.^13^ Lee et al also found the same finding in the United Kingdom with 1972 patients observed.^37^ *NRAS* mutation was significantly associated with tumor location, especially the extremities.^35^

In this study, we found no significant between the existence or lack of *NRAS* mutation and age, gender, and anatomical site. This result differs from the previous study by Sheen et al in Taiwan, which showed that *NRAS* mutation is associated with older age and caused by CSD. Several other factors that can trigger melanoma include geographical location, vitamin D deficiency, and unhealthy lifestyle habits such as smoking. Vitamin D deficiency may increase *NRAS* mutations. A study conducted in Indonesia showed that women of childbearing age (18‐40 years) had an average serum 25(OH)D level of 48 nmol/L with a prevalence of vitamin D deficiency of 63%. Women of childbearing age in Indonesia, who often work in the house, have a higher risk of low 25(OH)D serum levels. Habitual factors such as the use of sunscreens also affect the absorption of vitamin D by the skin, resulting in vitamin D deficiency, which can increase the occurrence of *NRAS* mutations in young women.^38^

This study revealed no statistically significant association between *NRAS* mutations and tumor size, mitotic index, ulceration, TIL, necrosis, LVI, and tumor thickness. This evidence is consistent with previous studies.^6^, ^20^ Moreno‐Ramírez et al study found that there was a moderate correlation between tumor size and Breslow thickness, but there are no studies linking tumor size with *NRAS* mutation status in patients with melanoma.^10^ The study by Jakob stated that this could occur since the data were collected retrospectively, which identified only patients with distant metastatic conditions, whereas this research focused on primary melanoma, specifically the cutaneous nodular subtype.^5^ Our study has some limitations because the small sample population was chosen retrospectively.

## 5 CONCLUSIONS

*NRAS* mutations were seen in 19.6% of primary nodular subtype of cutaneous melanoma cases in Yogyakarta and Central Java Province, Indonesia, which is consistent with data worldwide. We found the predominance of *NRAS* mutation in exon 2 (G12) in 60% of subjects, which is different from other studies. The existence of the *NRAS* mutation is significantly correlated with lymph node metastasis. Further study using state‐of‐the‐art methods such as next‐generation sequencing is needed to specify a more complete mutational profile of *NRAS* and other genes of the MAPK pathway in Asian populations, including Indonesian.

## CONFLICT OF INTEREST

The authors declare that they have no competing interests.

## AUTHOR CONTRIBUTIONS

*Conceptualization; data curation; formal analysis; funding acquisition; investigation; methodology; resources; software; supervision; validation; visualization; writing‐original draft; writing‐review & editing*, H.R.; *Data curation; formal analysis; investigation; methodology; project administration; validation; writing‐original draft; writing‐review & editing*, D.S. and F.T.; *Data curation; formal analysis; investigation; project administration; resources; supervision; validation; writing‐review & editing*, B.K.; *Formal analysis; investigation; methodology; supervision; validation; writing‐review & editing*, P.F.; *Formal analysis; investigation; methodology; resources; supervision; validation; writing‐review & editing*, I.

## ETHICS STATEMENT

The study protocol was reviewed and approved by the Medical and Health Research Ethics Committee of Faculty of Medicine, Public Health and Nursing, Universitas Gadjah Mada, Yogyakarta, Indonesia (letter‐number KE/FK/1016/EC/2019). Informed consent for usage of tissue samples for research was obtained preoperatively from the patients in written form.

## Data Availability

The data that support the findings of this study are available on request from the corresponding author upon reasonable request.
